# A Review of FDG-PET in Progressive Supranuclear Palsy and Corticobasal Syndrome

**DOI:** 10.3390/ijms26178278

**Published:** 2025-08-26

**Authors:** Alexandros Giannakis, Eugenia Kloufetou, Louisa Pechlivani, Chrissa Sioka, George Alexiou, Spiridon Konitsiotis, Athanassios P. Kyritsis

**Affiliations:** 1 Department of Neurology, Faculty of Medicine, School of Health Sciences, University Campus, University of Ioannina, 45500 Ioannina, Greece; papadates@gmail.com (A.G.); skonitso@uoi.gr (S.K.); 2 Neurosurgical Institute, University of Ioannina, 45500 Ioannina, Greece; louisapechlivani@gmail.com (L.P.); csioka@yahoo.com (C.S.); galexiou@uoi.gr (G.A.); 3 Department of Nuclear Medicine, Faculty of Medicine, School of Health Sciences, University Campus, University of Ioannina, 45500 Ioannina, Greece; 4 Department of Neurosurgery, Faculty of Medicine, School of Health Sciences, University Campus, University of Ioannina, 45500 Ioannina, Greece

**Keywords:** progressive supranuclear palsy, corticobasal syndrome, biomarker, differential diagnosis, positron emission tomography

## Abstract

Although diagnostic criteria and research are constantly advancing, distinguishing between progressive supranuclear palsy (PSP) and corticobasal syndrome (CBS) remains a significant challenge. This difficulty stems from their similar clinical symptoms and the lack of reliable biomarkers. In this work, we present a detailed review of fluorodeoxyglucose (FDG)–positron emission tomography (PET), exploring its potential role in differentiating PSP and CBS, drawing on their established utility in other neurodegenerative diseases. We searched the PubMed database from its inception for original research articles assessing the utility of FDG-PET for the diagnosis or differential diagnosis of PSP and CBS from other neurodegenerative conditions. A total of 91 studies were eligible. These 91 studies were categorized as follows: (a) 20 studies included only patients with PSP, (b) 15 studies included only patients with CBS, (c) 39 studies involved patients with Parkinson’s disease and atypical Parkinsonian disorders, including subgroups of PSP and/or CBS, and (d) 17 studies compared patients with PSP and/or CBS to individuals with Alzheimer’s disease, frontotemporal dementia, or other dementias. Most FDG-PET studies involving PSP and CBS were not specifically designed for these disorders. An additional obstacle lies in the methodological variability across studies. Despite several studies achieving high diagnostic accuracy for PSP and/or CBS with specificity exceeding 90% using FDG-PET, sensitivity remains considerably lower. CBS appears to have a distinct hypometabolic pattern compared to PSP, marked by asymmetry and predominant cortical involvement. CBS more often affects posterior cortical regions (parietal and posterior parts of the frontal cortex, and sometimes temporal and occipital parts) and the thalamus, whereas PSP appears to affect the striatum, frontal cortex, anterior cingulate, and subtentorial structures, typically in a more symmetrical manner. Large, multicenter studies are needed, utilizing standardized imaging and protocols.

## 1. Introduction

Progressive supranuclear palsy (PSP) and corticobasal syndrome (CBS) are rare, rapidly progressive neurodegenerative disorders that belong to the spectrum of atypical parkinsonian disorders (APDs). Both conditions are characterized by distinctive clinical features, considerable overlap with other neurodegenerative diseases, and complex underlying neuropathology [1,2]. PSP is classically associated with early postural instability, supranuclear gaze palsy, axial rigidity, and cognitive impairment, while CBS is often defined by asymmetric motor symptoms, apraxia, dystonia, and cortical sensory deficits [1,2]. Despite these somewhat characteristic features, clinical differentiation between PSP, CBS, and other neurodegenerative diseases remains challenging, particularly in early disease stages [3,4]. Additionally, other neurodegenerative diseases, such as Alzheimer’s disease (AD), further contribute to diagnostic complexity (15%) [5]. Moreover, emerging perspectives categorize CBS and PSP within broader and more heterogeneous neurodegenerative spectrums, such as frontotemporal dementia (FTD) and posterior cortical atrophy (PCA), further complicating clinical diagnosis [6,7].

As a result, accurate biomarkers that can improve diagnostic certainty and provide insights into disease mechanisms are of great clinical and research importance [8,9]. Taking the lead from other neurological disorders, where imaging biomarkers continue to emerge [10], reliable biomarkers in PSP and CBS are essential, especially since an effective treatment is still lacking [11].

One of the most widely studied imaging biomarkers in PSP and CBS is fluorodeoxyglucose–positron emission tomography (FDG-PET) [12,13]. FDG-PET provides a measure of regional cerebral glucose metabolism, serving as a proxy for synaptic activity and neuronal integrity [13]. In neurodegenerative disorders, patterns of hypometabolism often precede overt structural changes and can reveal disease-specific signatures that aid in differential diagnosis [14]. Over the past two decades, FDG-PET has emerged as a powerful tool to complement clinical assessment, structural magnetic resonance imaging (MRI), and other functional imaging modalities, such as tau- and amyloid-positron emission tomography (PET) [15].

This narrative review aims to provide a comprehensive overview of FDG-PET findings in PSP and CBS. By synthesizing the current evidence, this review seeks to clarify the utility and limitations of FDG-PET in PSP and CBS, and to identify directions for future research.

## 2. Results

### 2.1. FDG-PET in Progressive Supranuclear Palsy

Several studies have investigated the diagnostic utility of FDG-PET in PSP, either alone or in combination with CBS, with or without healthy controls for comparison. Table 1 summarizes the main findings of these studies.

Foster et al. used FDG-PET to study patients with PSP and compared them to healthy controls [16]. They observed reduced glucose metabolism in several subcortical structures—including the pons, thalamus, and caudate nucleus—but not in the cerebellum. In the cerebral cortex, multiple regions of interest (ROIs) showed hypometabolism, with the superior frontal cortex being the most severely affected. Notably, cortical hypometabolism was more pronounced than that observed in any subcortical region. Similarly, Goffinet et al. observed reduced glucose metabolism in the frontal lobe—particularly in the premotor and motor areas—of nine patients with PSP [17]. Karbe et al. also reported hypometabolism in the frontal lobe of PSP patients compared to normal controls [18]. However, they found more prominent hypometabolism in subcortical structures, including the upper midbrain, lentiform nucleus, and caudate nucleus—a pattern that differed slightly from that reported by Foster et al. The midbrain was the main ROI in the study of Mishina et al [21]. The researchers found hypometabolism in PSP patients compared to controls, independent of clinical deterioration.

Zhao et al. also reported decreased glucose metabolism in the midbrain, caudate nuclei, frontal lobes, and cingulate gyrus in patients with PSP [19]. Notably, midbrain hypometabolism was observed in 92.3% of PSP patients on FDG-PET, whereas only 57.9% showed the characteristic “hummingbird sign” in the same region on MRI. Yamauchi et al. combined FDG-PET and MRI to examine alterations in the corpus callosum of patients with PSP compared to healthy controls [20]. They observed corpus callosum atrophy on MRI in PSP patients, which correlated with frontal cortex hypometabolism, consistent with previous findings. Notably, these structural and metabolic changes were associated with poorer performance on neurocognitive tests of visuospatial ability and verbal fluency. Takahashi et al. compared MRI and FDG-PET in PSP vs. controls and found FDG-PET to be superior in detecting alterations in the middle frontal gyrus, medial/lateral frontal lobes, midbrain, and thalamus [22]. Notably, atrophy and hypometabolism of the frontal lobes were correlated with Mini-Mental State Examination (MMSE) scores.

Zwergal et al. also investigated hypometabolism in specific ROIs and its association with clinical feature severity in PSP [23]. Decreased FDG uptake in the thalami correlated with fall frequency, while increased metabolism in the precentral gyrus was also linked to increased fall risk. Subsequently, they showed that gait severity scales were correlated with hypometabolism in the prefrontal cortex, subthalamic nucleus, and pedunculopontine/cuneiform nucleus complex—findings that were confirmed both at rest and after walking [24]. Similarly, Amtage et al. examined gaze palsy patterns and their association with focal brain hypometabolism in PSP patients [25]. They found that downward gaze palsy correlated with bilateral hypometabolism in the anterior cingulate and the right lingual gyrus. On the other hand, Isella et al. focused on phonemic fluency in PSP and found that patients with reduced phonemic fluency exhibited decreased glucose metabolism in the dominant superior frontal cortex [26]. Doll-Lee et al. investigated cognitive impairment and cerebral glucose metabolism in patients with PSP [27]. Compared to healthy controls, PSP patients demonstrated reduced FDG uptake in the left inferior frontal gyrus and right angular gyrus. Notably, overall cognitive performance was correlated with metabolic alterations in the right frontal eye field. In addition, list learning was associated with metabolism in the left frontal eye field, while verbal fluency correlated with activity in the bilateral premotor and supplementary motor cortices.

Data on FDG-PET performance in specific PSP subtypes remains limited. Buchert et al. recruited patients with PSP–Richardson syndrome (PSP-RS) and other PSP subtypes, comparing them to healthy controls [28]. They evaluated visual analysis supported by voxel-based statistics alongside automatic covariance pattern analysis. While visual analysis demonstrated only modest sensitivity and specificity, performance improved significantly with automatic analysis. Notably, the rate of false-negative cases decreased overall and was reduced to zero specifically in the PSP-RS group. Subsequently, they employed MRI volumetric analysis, which demonstrated a comparable area under the curve (AUC) to that of automated FDG-PET analysis, but with higher specificity and lower sensitivity [35]. Black et al. compared metabolic patterns across the prefrontal, premotor, and sensorimotor cortices in 137 patients with various PSP subtypes, including PSP with predominant speech/language impairment (PSP-SL), corticobasal syndrome (PSP-CBS), frontal presentation (PSP-F), Parkinsonism (PSP-P), and progressive gait freezing (PSP-PGF) [30]. Notably, 43 patients underwent autopsy. They found frontal hypometabolism in 100% of patients with PSP-SL, PSP-CBS, and PSP-F, whereas lower rates were observed in other subtypes, such as 73% in PSP-PGF. Hypometabolism was most frequently asymmetric in the PSP-CBS, PSP-SL, PSP-P, and PSP-F groups. Additionally, Frontal Assessment Battery (FAB) scores were negatively correlated with glucose metabolism across all subtypes.

Garraux et al. were among the first to compare patients with PSP and CBD [29]. Compared to healthy controls, patients with CBD showed asymmetric hypometabolism affecting the putamen, thalamus, precentral gyrus, lateral premotor cortex, supplementary motor area, dorsolateral prefrontal cortex, and inferior parietal cortex. In contrast, when directly compared to PSP, CBD patients exhibited more posterior hypometabolism, including in the supplementary motor area. PSP patients, on the other hand, demonstrated more prominent hypometabolism in the midbrain, anterior cingulate cortex, and orbitofrontal cortex. Similarly, Hosaka et al. PSP showed hypometabolism in the medial and lateral frontal gyri, along with midbrain and basal ganglia alterations [31]. In contrast, CBS showed predominant parietal lobe hypometabolism, compared to both controls and PSP. Juh et al. conducted a study comparing 8 patients with CBD and 8 with PSP to 22 healthy controls [32]. When comparing the two patient groups directly, they found hypometabolism in the midbrain and thalamus in the PSP group, and in the parietal lobe in the CBD group. In addition, compared to controls, PSP patients exhibited hypometabolism in the orbitofrontal cortex, middle frontal cortex, and cingulate gyrus, while CBD patients showed asymmetric hypometabolism in the frontal and cingulate cortices, in addition to the parietal lobe involvement mentioned earlier.

Amtage et al. also compared PSP patients with either unilateral or bilateral motor impairment to patients with CBD and found similar hypometabolic patterns [33]. Interestingly, PSP patients with unilateral motor impairment showed hypometabolism in the contralateral thalamus, middle cingulate gyrus, and sensorimotor cortex compared to controls. Zalewski et al. evaluated FDG-PET findings in seven patients with autopsy-confirmed PSP, two cases of globular glial tauopathy (GGT), and one case of CBD [34]. They observed bilateral hypometabolism in the caudate, thalamus, and midbrain across all cases. No significant differences were found between the PSP and GGT cases, whereas the CBD case demonstrated more prominent and asymmetric hypometabolism, particularly in the parietal lobes.

**Figure 1:**
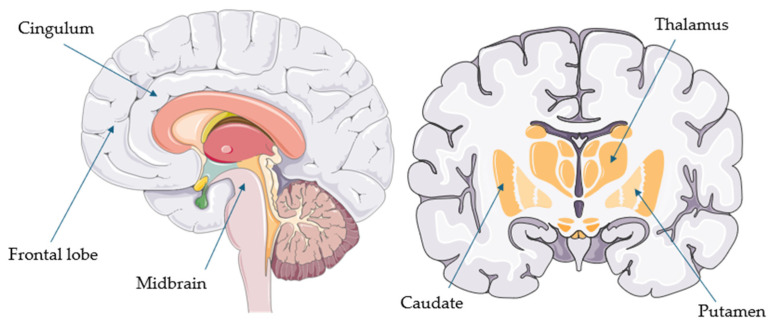
Common hypometabolic regions in progressive supranuclear palsy (parts of the image provided by Servier Medical Art (https://smart.servier.com/ (accessed on 21 August 2025)), licensed under CC BY 4.0 (https://creativecommons.org/licenses/by/4.0/ (accessed on 21 August 2025)).

Table 2 summarizes the main hypometabolic regions reported in each PSP study (when available). These regions are also illustrated in Figure 1.

### 2.2. FDG-PET in Corticobasal Syndrome

Table 3 summarizes the main findings that utilize FDG-PET in CBS patients, either alone or in comparison with controls.

Blin et al. initially studied five patients with a clinical diagnosis of CBD and compared them to healthy controls [36]. Although most cortical and subcortical regions showed reduced metabolism on FDG-PET in the CBD group, the greatest reductions in glucose uptake were observed in the temporal and sensorimotor cortices contralateral to the most affected side.

Multiple studies have compared FDG-PET with other imaging modalities in patients with CBS. Klaffke et al. reported contralateral hypometabolism in multiple cortical and subcortical regions in patients with CBD, while ^123^I-iodobenzamide (IBZM) SPECT imaging revealed reduced dopamine D2 receptor binding in only one patient, suggesting limited sensitivity of IBZM in detecting basal ganglia involvement in CBD [37]. Turaga et al. also observed asymmetrical hypometabolism involving the left basal ganglia and the inferior parietal, temporal, and frontal lobes [38]. These metabolic findings correlated with MRI-detected atrophy in the left temporal and parietal cortex as well as the basal ganglia—though notably, not in the parietal cortex—suggesting greater sensitivity of FDG-PET for detecting early or subtle changes. Importantly, FDG-PET abnormalities also correlated with deficits in neurocognitive assessments, particularly in frontal/executive function, speech, and visuospatial abilities. Franceschi et al. also employed FDG-PET and volumetric MRI to retrospectively assess patients with clinically established dementia [39]. Of the 12 patients later suspected to have CBD, 10 exhibited the previously described asymmetrical hypometabolic pattern involving both cortical and subcortical regions. This pattern corresponded to atrophy in the same areas on volumetric MRI. The remaining two patients demonstrated a hypometabolic pattern affecting the sensorimotor cortex bilaterally.

Sha et al. employed both FDG-PET and amyloid PET in a cohort of 25 patients with CBS, 8 of whom later had autopsy-confirmed diagnoses [40]. FDG-PET demonstrated higher sensitivity than amyloid PET in detecting CBS, but with lower specificity. On the other hand, Mille et al. combined FDG-PET and dopamine transporter imaging with single-photon emission computed tomography (DAT-SPECT) to assess CBS patients and found that, despite the hypometabolism of the frontal and parietal association cortices and putamen contralateral to most severely motor affected side, DAT-SPECT showed only slight asymmetry in DAT availability [51].

Isella et al. analyzed 35 patients with CBD and identified hypometabolism in the left inferior parietal, primary motor, primary sensory, and insular cortices [42]. Furthermore, they found an association between these hypometabolic ROIs and patients’ cognitive reserve, as measured by years of education. They also found that patients with CBS and asymmetric hypometabolism in the left hemisphere showed greater deficits in digit span, whereas those with right-hemisphere hypometabolism exhibited more pronounced visuospatial impairments [43].

Pardini et al. conducted one of the few studies involving only CBS patients with autopsy-confirmed diagnoses [44]. All patients showed hypometabolism in the perirolandic area, basal ganglia, and thalamus contralateral to the most affected side. Notably, patients with clinical CBS and CBD pathology demonstrated more pronounced and bilateral basal ganglia involvement, whereas those with underlying AD pathology exhibited more asymmetric hypometabolism in the lateral parietal and temporal lobes, as well as the posterior cingulate. In contrast, patients with PSP pathology had greater involvement of the anterior cingulate and medial frontal cortex. These findings suggest that distinct FDG-PET hypometabolic patterns in CBS may help differentiate the underlying pathology.

Data on the application of FDG-PET across specific subtypes of CBD or certain disease characteristics remain limited. Jo et al. investigated hypometabolic patterns in 52 patients with CBD by categorizing them into groups based on the presence of ideomotor apraxia, imitation apraxia, or both, and compared them to healthy controls [45]. Notably, patients with ideomotor apraxia were more likely to exhibit hypometabolism in the left angular gyrus, whereas those with imitation apraxia more commonly showed hypometabolism in the precuneus, postcentral gyrus, and posterior cingulate cortex. CBD was also associated with hypometabolism of the frontal gyrus and caudate, regardless of the apraxia. Parmera et al. employed FDG-PET, amyloid PET, and MRI with voxel-based morphometry to examine patients with probable CBS and specific neurocognitive deficits [46]. Interestingly, patients presenting with dysarthria showed hypometabolism in the left inferior frontal gyrus and premotor cortex, while MRI revealed pronounced atrophy in the frontal operculum and putamen. In contrast, phonemic fluency was positively correlated with glucose uptake in the frontal operculum as well as the inferior and middle temporal gyri, whereas semantic fluency correlated with glucose uptake in the lateral temporal lobe. Subsequently, they combined FDG-PET and amyloid PET imaging in patients with CBS, further categorizing them based on whether they fulfilled the Movement Disorder Society (MDS) criteria for 4R tauopathies. Interestingly, patients who met the criteria exhibited a distinct hypometabolic pattern involving the supplementary motor area, bilateral striatum, and anterior cingulate cortex—unlike those who did not meet the criteria [47]. Moreover, Nakano et al. combined FDG-PET, amyloid PET, and tau PET to investigate patients with CBS [49]. They found that patients with positive tau PET and negative amyloid PET expressed decreased glucose uptake in the precentral gyrus and thalamus compared to controls. Parmera et al. combined FDG-PET and amyloid PET to investigate patients with probable CBS [48]. They found that worse cognitive performance was most common in patients exhibiting an AD-like hypometabolic pattern on FDG-PET, whereas greater motor impairment was more often associated with a CBD-like pattern. Moreover, FDG-PET demonstrated very high specificity and positive predictive value, but only modest sensitivity in predicting amyloid positivity on amyloid PET based on its metabolic classification.

Similarly, Ghirelli et al. combined FDG-PET, tau PET, and amyloid PET to examine the association between tau and amyloid positivity and hypometabolism in patients with CBS [50]. They found that patients positive for both amyloid and tau exhibited more extensive hypometabolism in the lateral temporal, parietal, and occipital lobes compared to those who were tau-negative and either amyloid-positive or amyloid-negative. Additionally, this group showed more pronounced asymmetry, whereas metabolic patterns in the mesial temporal lobes and basal ganglia did not differ significantly across the three groups

**Figure 2:**
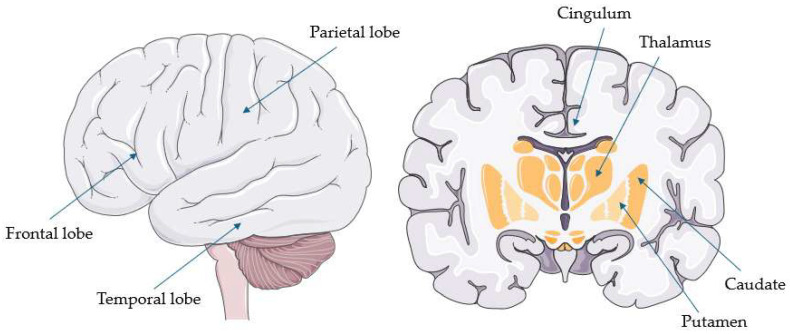
Common hypometabolic regions in corticobasal syndrome (parts of the image provided by Servier Medical Art (https://smart.servier.com/ accessed on 21 August 2025), licensed under CC BY 4.0 (https://creativecommons.org/licenses/by/4.0/ accessed on 21 August 2025).

Table 4 summarizes the main hypometabolic regions reported in each PSP study (when available). These regions are also illustrated in Figure 2.

### 2.3. FDG-PET in Atypical Parkinsonian Disorders

FDG-PET has been extensively studied in Parkinson’s disease PD and APDs, with recommendations on its diagnostic utility and interpretation of findings [12]. Table 5 provides an overview of key studies comparing PSP and/or CBS with other Parkinsonian disorders.

Eckert et al. used FDG-PET to examine hypometabolic patterns in patients with PSP, patients with multiple system atrophy (MSA), and healthy controls [52]. Their findings revealed a distinct hypometabolic pattern in PSP, primarily affecting the brainstem and bilateral medial frontal cortex. In contrast, MSA exhibited hypometabolism predominantly in the putamen and cerebellum. These characteristic patterns effectively distinguished both diseases from healthy controls (*p* < 0.001). Mudali et al. compared FDG-PET patterns among patients with PSP, PD, and MSA, as well as healthy controls [53]. Their computational analysis distinguished PSP from controls with moderate accuracy (AUC = 0.8) but showed relatively low accuracy in differentiating PSP from PD (AUC = 67.6) and MSA (AUC = 68.4).

Furthermore, Srulijes et al. compared FDG-PET patterns among patients with PSP-RS, PSP-P, and PD, as well as healthy controls [54]. They found thalamic hypometabolism in PSP-RS and putaminal hypometabolism in PSP-P compared to all other groups. Additionally, PSP-RS exhibited frontal hypometabolism. The putamen/thalamus uptake ratio effectively differentiated PSP-P from PSP-RS with good accuracy (AUC = 0.86) and from PD with an AUC of 0.80. Eckert et al. compared clinical and computerized assessments of patients with PSP, CBD, PD, and MSA, finding a high agreement rate of 92.4% [55]. Botha et al. investigated midbrain hypometabolism, known as the “pimple sign”, in PSP compared to CBS and MSA [90]. Their findings showed high specificity for PSP (100%), but very low sensitivity (29%)

Niethammer et al. investigated a pattern of bilateral, asymmetric hypometabolic dysfunction in the frontal and parietal cortices, as well as the caudate nuclei and thalami [56]. Their analysis revealed a significant overlap (24%) between CBD and PSP patients, but good discriminating ability between CBD and MSA. However, a logistic algorithm incorporating asymmetry scores and a distinct PSP pattern achieved high specificity for both CBD (92.7%) and PSP (94.1%). Similarly, in a follow-up period of 9 months, Hellwig et al. were able to discriminate PSP, CBD, PD, and MSA by utilizing distinctive hypometabolic patterns for each disease, achieving 90% diagnostic accuracy [57]. Comparing the same patient groups, Tripathi et al. achieved a high concordance between clinical and FDG-PET diagnoses (90.4% for PD, 80% for MSA, 93.3% for PSP, and 100% for CBS). Subsequently, Hellwig et al. have demonstrated moderate specificity and high sensitivity in diagnosing PSP (74% and 95%, respectively) and CBD (75% and 92%, respectively) in a comparative study with PD, dementia with Lewy bodies (DLB), and MSA patients [59]. In a reverse approach, Tang et al. enrolled patients with Parkinsonism and categorized them into PSP, PD, and MSA after calculating hypometabolic patterns based on logistic regression [60]. They then followed up with the patients under the care of a movement disorders specialist. PSP was predicted with 88% sensitivity and 94% specificity

Juh et al. compared patterns of hypometabolism in patients diagnosed with PSP, PD, and MSA, as well as in healthy controls [61]. All disease groups exhibited reduced metabolic activity in the neocortex. However, PSP was uniquely characterized by significant hypometabolism extending to subcortical regions, specifically the caudate nucleus, thalamus, midbrain, and anterior cingulum. These findings differentiated PSP not only from healthy controls but also from PD and MSA, suggesting distinct neuropathological processes.

Garraux et al. also utilized FDG-PET to compare patients with PSP, CBS, MSA, and PD [62]. However, classification accuracy was low for all disease study groups. Tripathi et al. continued their previous research by developing an algorithm with 94% specificity for differentiating PSP from PD and MSA [63]. Brajkovic et al. used FDG-PET hypometabolism to initially diagnose Parkinsonian syndromes [64]. After two years of clinical follow-up by a movement disorders specialist, the FDG-PET diagnosis was confirmed in 97% of PSP cases and 100% of CBS cases, compared to PD and MSA.

Furthermore, Marti-Andres et al. analyzed FDG-PET patterns in PSP subtypes, i.e., PSP-RS, PSP-P, PSP-PGF, PD, and controls [65]. PSP was distinguished from controls with 80% sensitivity and 96.9% specificity, and from PD with 80.4% sensitivity and 90.7% specificity. Notably, PSP-RS and PSP-P exhibited PSP-like hypometabolism more often than PD [65].

Shen et al. applied an MSA-defined FDG-PET hypometabolic pattern, involving decreased metabolism in the inferior frontal cortex, striatum, and cerebellum and increased metabolism in the occipital, parietal, and sensorimotor cortices, to differentiate patients with PSP, PD, and MSA from healthy controls [66]. The study demonstrated successful discrimination between all groups, highlighting the pattern’s diagnostic potential [66].

Moreover, Amod et al. employed FDG-PET to distinguish between patients with PSP, CBS, PD, MSA, and DLB, as well as healthy controls [67]. The study accurately identified 90% of PD patients and 93% of patients with PSP and CBS [67]. Lastly, Tomse et al. utilized PSP- and MSA-related pattern to compare patients with PSP, MSA—Parkinsonian type (MSA-P), and PD, as well as healthy controls. Both patterns were highly sensitive and specific (AUC 0.99 and 0.96, respectively) [68].

Eidelberg et al. were among the first to utilize FDG-PET to compare patients with CBD, patients with PD, and healthy controls [69]. Their findings revealed that the CBD group exhibited more pronounced and asymmetric hypometabolism in the thalamus, inferior parietal lobule, and hippocampus compared to the other groups [69]. Karbe et al. were among the first to compare patients with PSP, PD, and Parkinsonism (“PD plus dementia of Alzheimer type”) [70]. The latter group exhibited diffuse cortical hypometabolism, most prominently in the parietal lobes, whereas PD patients showed no significant cortical hypometabolism. In contrast, PSP patients demonstrated hypometabolism in the brainstem, putamen, caudate, and frontal cortex [70].

Klein et al. also compared PSP and PD patients. PSP patients showed hypometabolism in the dorsal midbrain and the caudal anterior cingulate, whereas PD patients exhibited hypometabolism in the lateral visual cortex and the right fusiform gyrus [71].

Herting et al. examined the relationship between brain hypometabolism and depression in patients with PSP, patients with MSA, and healthy controls. In PSP patients, reduced glucose metabolism was observed in the bilateral frontal cortex, right thalamus, and midbrain [72]. Additionally, in both PSP and MSA, depression severity was positively associated with hypometabolism in the dorsolateral prefrontal cortex. MSA patients also demonstrated bilateral hypometabolism in the cerebellar, frontal, and parietal cortices, as well as in the left putamen [72].

Park et al. compared patients with pure akinesia with gait freezing (PAGF) to those with PSP and PD, as well as healthy controls [73]. They found that PAGF patients exhibited hypometabolism in the midbrain, whereas PSP patients showed hypometabolism in both the midbrain and frontal cortex, consistent with prior findings. These results suggest one of the first in vivo pathophysiological links between PAGF and PSP, as demonstrated by FDG-PET imaging [73]. Likewise, in a notable prospective study, Josephs et al. followed 13 patients with primary progressive apraxia of speech (PPAOS) and found that 8 later progressed to PSP [78]. All patients demonstrated an expansion of their hypometabolic patterns over time [78].

Hellwig et al. sought to differentiate major neurodegenerative parkinsonian syndromes by combining FDG-PET and IBZM-SPECT imaging [74]. Their approach aimed to distinguish between PD, PD dementia (PDD), PSP, CBD, DLB, and MSA. The combined imaging yielded a sensitivity of 74% and specificity of 95% for PSP, and 75% sensitivity with 92% specificity for CBD. Notably, IBZM-SPECT was less effective than FDG-PET in distinguishing Lewy body disorders (PD, PDD, and DLB) from APDs [74]. Similarly, Teune et al. employed multivariate covariance analysis of FDG-PET data and demonstrated high discriminative accuracy for differentiating PSP, PD, and MSA based on their distinct metabolic patterns, even in early disease stages [75]. These findings were validated by long-term clinical follow-up confirming the diagnoses. However, Akdemir et al., using both visual and quantitative FDG-PET analysis, reported more heterogeneous results [76]. Most patients with PD, PSP, CBD, DLB, and MSA showed basal ganglia hypometabolism, with asymmetric patterns more frequently observed in PSP and PD. Additionally, some PSP and CBD patients exhibited thalamic hypometabolism, while cerebellar hypometabolism was predominant among MSA patients [76]. On the other hand, Baudrexel et al. combined FDG-PET with MRI diffusivity sequences and found that hypometabolism of the posterior putamen was associated with increased mean diffusivity in patients with MSA-P [77]. However, both imaging modalities demonstrated comparable discriminatory power in differentiating patients with PSP, patients with PD, and healthy controls [77].

Ko et al. combined FDG-PET and DAT-SPECT imaging to differentiate between PSP, CBS, PD, MSA, and DLB patients [79]. They found that metabolic and DAT binding patterns were correlated in CBS and PD, but not in PSP or MSA.

In addition, Ge et al. recruited patients with PSP, PD, and MSA from cohorts in both China and the United States [80]. They identified a characteristic hypometabolic pattern involving the cingulate, ventrolateral and middle prefrontal cortex, striatum, thalamus, and midbrain, along with relative hypermetabolism in the hippocampus and temporoparietal regions. This pattern effectively distinguished PSP from other groups with high reproducibility [80]. Similarly, Arnone et al. demonstrated that hypometabolic and hypermetabolic pattern maps improved diagnostic accuracy for non-experts when assessing patients with PSP, CBS, PD, and MSA [81]. In contrast, for expert clinicians, only the hypometabolic maps significantly enhanced diagnostic accuracy [81].

Lu et al. utilized two of the largest patient cohorts with Parkinsonism and FDG-PET and applied algorithms along with age- and gender-specific Z-scores to adjust for metabolic changes [82]. This approach achieved high diagnostic accuracy in distinguishing PSP, PD, and MSA [82].

On the other hand, in the study by Ouartassi et al., only half of the CBS patients exhibited a CBD-like hypometabolic pattern when compared to patients with PSP, patients with PD, and healthy controls [83]. In the study by Ali et al., FDG-PET was combined with volumetric MRI in patients with PSP, including both PSP-RS and other subtypes [84]. Clinical assessments included the Saccadic Impairment Scale (SIS) for vertical gaze palsy, the Montreal Cognitive Assessment (MoCA) for global cognition, and the MDS-Unified Parkinson’s Disease Rating Scale (MDS-UPDRS). The findings showed that both imaging modalities were more effective at predicting the presence of specific clinical features rather than their severity, suggesting their greater utility in assessing the state of the disease rather than its stage. FDG-PET was more sensitive to cortical-related clinical features, such as apraxia and executive dysfunction, while volumetric MRI was more effective in detecting subcortical-related features, such as Parkinsonism. The strongest predictive power for both modalities was observed for global cognitive performance as measured by MoCA. Furthermore, for specific symptoms, the imaging modality best predicted the corresponding anatomical substrates—for example, midbrain atrophy on MRI and frontal eye field hypometabolism on FDG-PET were both associated with vertical gaze palsy [84].

In a study by Du et al., the use of automated assessment tools—achieved very high diagnostic accuracy in differentiating patients with PD from those with PSP [85]. The study also included patients with MSA-P and vascular Parkinsonism [85]. Similarly, Ling et al. utilized a radiomics-guided deep learning model to differentiate patients from a large cohort with PD, PSP, MSA, and controls, achieving high sensitivity across all groups [86]. Subsequently, they applied a metabolic framework to the same cohort, which not only achieved high classification performance across all patient groups but also revealed disease-specific hypometabolic ROIs and networks—e.g., midbrain and midbrain-prefrontal disconnection in PSP [87]. Pillai et al. combined multiple MRI modalities with FDG-PET to study distinct PSP subtypes and patients with PD, demonstrating that FDG-PET could effectively differentiate PSP from PD [88]. Lastly, Stokelj et al. utilized FDG-PET data to develop an algorithm for differentiating patients with PD, PSP, and MSA [89]. However, while they achieved a high AUC for PSP, the correct identification rate of PSP patients was modest [89].

### 2.4. FDG-PET in Progressive Supranuclear Palsy and/or Corticobasal Syndrome Compared to Frontotemporal Dementia, Alzheimer’s Disease, and/or Neurodegenerative Diseases

FDG-PET has also been studied in FTD syndromes, including PSP and CBS. Table 6 provides an overview of key studies comparing PSP and/or CBS with other types of FTD, as well as AD and other neurodegenerative diseases.

Cerami et al. analyzed FDG-PET metabolic patterns in a large cohort of PPA patients [91]. This identified key ROIs associated with language impairment, including the left temporal (inferior, middle, and superior gyri, and pole), frontal (superior, middle, and inferior gyri), precentral gyrus, and parietal (inferior and superior lobules) regions. Specific hypometabolic patterns were observed for each PPA variant. Patients later diagnosed with CBD showed asymmetric parietal hypometabolism, while those meeting PSP criteria involved the midbrain and cerebellum. lvPPA patients presented with hypometabolism in the left temporal and parietal regions, with hippocampal involvement in 65% of cases. Clinical follow-up revealed progression to FTD in 6/11 svPPA patients, AD in 10/17 lvPPA patients, CBD in 11/19 nfvPPA patients, and PSP in 3/19 nfvPPA patients [91]. The same study group also stratified CBS patients by AD-related CSF biomarkers. Positive biomarkers correlated with typical AD hypometabolism (precuneus, posterior cingulate, and temporoparietal). Negative biomarkers correlated with bilateral frontoinsular and basal ganglia hypometabolism [92]. Similarly, Caminiti et al. studied the phenoconversion of MCI to AD and other dementias [14]. During clinical follow-up, 50 out of 80 MCI cases progressed to dementia, with 39 converting to AD dementia, 10 to FTD (including 2 to CBD and 1 to PSP), and 1 to DLB [14].

David Bergeron et al. have shown that, in patients with non-amnestic variants of AD and FTD, the dominant middle temporal gyrus showed the most hypometabolism in lvPPA, while the middle occipital gyrus showed the most hypometabolism in PCA, the middle temporal gyrus showed the most hypometabolism in frontal AD, and the angular gyrus showed the most hypometabolism in CBS associated with AD pathology [93].

Heikkinen et al. aimed to differentiate PSP, CBD, and bvFTD patients with extrapyramidal symptoms from those without extrapyramidal symptoms using FDG-PET and MRI biomarkers [94]. Patients with extrapyramidal symptoms demonstrated predominant hypometabolism in the left hemisphere, particularly in the temporal and medial frontal lobes. More specifically, in bvFTD, patients exhibited metabolic changes in the left temporal lobe, along with hypometabolism in the superior cerebellar peduncle, cerebellar lingula, and frontal lobes. In contrast, no cerebellar hypometabolism was observed in PSP or CBD [94]. Similarly, Isella et al. utilized FDG-PET to study hypometabolism in the precuneus, posterior cingulate, and temporoparietal cortex in cognitively impaired patients with a suspected underlying neurodegenerative cause [95]. Patients were divided into AD biomarker-positive and biomarker-negative groups, with metabolism estimated either visually or using a semi-quantitative tool. Interestingly, cases with an asymmetric temporoparietal profile and a CBS or PPA phenotype were more frequently classified as having an AD-like pattern, even in the absence of other AD biomarkers [95].

Teune et al. recruited early-stage patients with PSP, CBD, PD, DLB, MSA, AD, and FTD, and successfully discriminated between these conditions by analyzing disease-specific hypometabolic patterns—such as in the prefrontal cortex, caudate, thalamus, and midbrain for PSP, and the contralateral cortex for CBD [96]. However, when Wenzel et al. applied a b-spline method for stereotactic normalization of FDG-PET data from patients with CBD, AD, DLB, and FTD, as well as healthy controls, it had an insignificant impact on expert-based classification outcomes [97].

Garibotto et al. combined FDG-PET with DAT-SPECT to differentiate patients with CBD, AD, DLB, and PDD, and found that the combined approach offered greater diagnostic accuracy than either modality alone, both in discriminant analysis and cross-validation [98]. Similarly, Taswell et al. combined FDG-PET with amyloid PET to distinguish between patients with CBD, AD, lvPPA, nfvPPA, and svPPA, reporting that FDG-PET outperformed clinical assessment in the diagnostic accuracy of AD [99]. Moreover, Franceschi et al. combined FDG-PET with volumetric MRI to study patients with CBD, AD, DLB, and FTD [100]. They found a Pearson correlation coefficient of 0.58 (*p* < 0.05) between the metabolic z-score and lobar volume in the superior parietal lobule for CBD patients [100]. Subsequently, the same study group used the same imaging combination to investigate crossed cerebellar diaschisis in patients with neurodegenerative diseases. However, only 7.5% of patients exhibited crossed cerebellar diaschisis, three of whom showed imaging findings consistent with CBD [101]. They also applied the same modalities to patients within the FTLD and PPA spectrum. In these cases, patients with PSP showed hypometabolism in the posterior frontal cortex, thalamus, basal ganglia, and midbrain. In contrast, patients with CBD exhibited predominantly asymmetric hypometabolism in the sensorimotor cortex, basal ganglia, and thalamus [102].

On the other hand, Josephs et al. followed patients with PPAOS over a period of more than ten years, all of whom underwent autopsy. The majority were ultimately diagnosed with either PSP or CBD [103]. Interestingly, FDG-PET scans at the initial evaluation showed no significant metabolic differences between patients who later developed PSP or CBD. However, after four years, patients with CBD exhibited greater rates of metabolic decline and lower cortical metabolism. Performance on neurocognitive tests correlated with metabolism in the left Broca’s area in both groups, and additionally with the left superior temporal gyrus in the CBD group [103]. However, when Seckin et al. followed up patients with PPAOS, all of whom eventually developed either PSP or CBS, they observed only slight left-sided asymmetric hypometabolism in the CBS group, and absent or minimal midbrain hypometabolism in the PSP group [104].

Gan et al. applied FDG-PET in patients with various dementia syndromes, including PSP and CBS, and found that patients within the FTLD spectrum, including PSP and CBS, generally showed modest percentages of frontal and anterior temporal hypometabolism [105]. Lastly, Braun et al. combined FDG-PET with MRI to distinguish autopsy-confirmed PSP cases from a diverse cohort of neurodegenerative diseases—including CBD, AD, PCA, PPAOS, PD, DLB, MSA, and Pick’s disease—achieving high classification accuracy [106].

## 3. Discussion

Overall, FDG-PET appears to be a useful supportive biomarker for the diagnosis of PSP and CBS. This aligns with the latest European intersocietal recommendations, which suggest that FDG-PET should be the first assessment tool once a clinical syndrome of PSP or CBS has been recognized [13]. In PSP, the most common hypometabolic regions include the midbrain (particularly the tegmentum) and extensive areas of the frontal cortex (such as the sensorimotor, premotor, orbitofrontal, prefrontal, superior, middle, inferior, and medial regions), usually bilaterally [30,32]. These are followed by the cingulate cortex (especially the anterior cingulum), caudate, putamen, and thalamus [21]. Other regions—such as the globus pallidus, parietal and insular cortices, pons, cerebellar vermis, and superior cerebellar peduncles—are less commonly involved [16,21,24,27,29].

CBS appears to have a hypometabolic pattern that is not commonly encountered in PSP. Firstly, unlike PSP, hypometabolism in CBS is highly asymmetrical, predominantly affecting the hemisphere contralateral to the most clinically affected side [23,44,47]. Secondly, cortical and lobar structures appear to be more commonly affected than subcortical or subtentorial regions. Indeed, in contrast to PSP, hypometabolism of the midbrain or other subtentorial structures is only rarely reported [21,50], while among subcortical but supratentorial regions, the thalamus appears to be more frequently involved than the striatum [37,48,49]. Thirdly, whereas PSP more commonly affects the frontal cortex, CBS often extends to more posterior cortical regions, particularly the parietal lobe (especially the postcentral, inferior, and superior parietal gyri), and less frequently the lateral temporal and occipital cortices—areas rarely affected in PSP [26,42,44,45,46,48]. Moreover, when the frontal lobe is implicated in CBS, its more posterior regions (e.g., precentral gyrus, supplementary motor area, and inferior frontal gyrus) are typically involved, whereas, in PSP, frontal hypometabolism tends to be more generalized [26,42,44,45,48]. Similarly, while PSP more often affects the anterior cingulate, CBS also involves its posterior regions [23,24,45,47].

However, several methodological and interpretative issues are evident in many of the studies included in this review. The majority of FDG-PET studies involving PSP and CBS were not specifically designed for these disorders. Instead, their primary aim was to differentiate PD from APDs, which simply include PSP and CBS. As a result, PSP and CBS data were often grouped with other APDs, providing extensive comparative data between these disorders and PD but limiting direct comparisons with other Parkinsonian syndromes, such as MSA and DLB. This lack of direct comparison hinders efforts to achieve nuanced differential diagnoses.

A similar issue arises when comparing PSP and CBS to FTLD spectrum disorders, such as bvFTD and PPA. The substantial clinical overlap between these syndromes complicates biomarker studies. Current diagnostic criteria acknowledge that bvFTD and PPA can manifest as clinical presentations of underlying PSP or CBS pathology [1,2].

Therefore, studies including only autopsy-confirmed cases are essential to avoid grouping patients who share the same clinical syndrome but have different underlying pathologies. Nonetheless, even autopsy-confirmed cases may still be affected by selection bias [107].

Moreover, the methodology varies significantly, with studies using different FDG protocols or applying distinct FDG patterns associated with various neurodegenerative diseases [108]. Furthermore, these studies often begin with different baseline cognitive or neurodegenerative syndromes (e.g., MCI or PPA) and examine how they progress into more specific syndromes, such as PSP and CBS, over diverse follow-up periods. This variability is common in studies enrolling patients with FTD syndromes. Additionally, while some studies incorporate follow-up with repeated FDG-PET imaging, others rely solely on clinical re-evaluation. These methodological discrepancies greatly affect the generalizability of the results [109].

Furthermore, only studies with generally small sample sizes specifically focus on PSP and/or CBS subtypes [30,88,106], which exhibit distinct clinical courses and prognoses. Some subtypes may also benefit from different symptomatic treatment approaches, highlighting the need for more targeted research in this area. In addition, only a few studies validate biomarker- or clinically based diagnoses through autopsy, which remains the gold standard for confirming PSP and CBS. The lack of postmortem validation limits the ability to assess the true diagnostic accuracy of these approaches and underscores the need for more studies incorporating neuropathological confirmation.

Despite several studies achieving high diagnostic accuracy for PSP and/or CBS with specificity exceeding 90% using FDG-PET, sensitivity remains considerably lower [65,74,90]. Moreover, high specificity is more frequently attained with the aid of quantitative or semiquantitative computational analysis, whereas simple visual evaluation yields more modest results. Thus, computer-assisted analysis appears to provide high specificity, suggesting that FDG-PET could be a valuable tool in the differential diagnosis of PSP and CBS. Such a combination of fluid and imaging biomarkers may offer a more com-prehensive diagnostic approach while reducing the need for more invasive procedures, such as lumbar puncture for CSF biomarker analysis. A large-scale, multicenter cohort study specifically focused on PSP and CBS, incorporating both FDG-PET and serum biomarkers, could help to establish a more accurate diagnostic framework for these overlapping syndromes.

In addition, a combination of FDG-PET with other emerging techniques may boost its diagnostic utility. For example, combining FDG-PET with tau PET imaging may increase the diagnostic accuracy and understanding PSP and CBS. These imaging modalities provide complementary insights: tau PET identifies the distribution and burden of tau protein deposits, while FDG-PET assesses regional cerebral glucose metabolism, reflecting neuronal activity and integrity [110]. Thus, in PSP, tau PET imaging has demonstrated increased uptake in certain regions, such as the midbrain, thalamus, and basal ganglia, corresponding to areas affected by tau pathology [110,111]. FDG-PET in PSP typically shows hypometabolism in the frontal lobes, midbrain, and caudate nucleus, aligning with the regions of tau deposition [110,112]. The combination of these imaging techniques allows for a more comprehensive assessment of both the distribution of tau pathology and the functional consequences of neuronal degeneration [112].

Similarly, in CBS, tau PET imaging has revealed asymmetric tau deposition, particularly in the parietal and frontal cortices, which are associated with the clinical manifestations of the syndrome [113]. FDG-PET in CBS often shows hypometabolism in the affected cortical regions, correlating with the areas of tau accumulation [110,113,114]. The integration of tau PET and FDG-PET provides a dual perspective on the structural and functional abnormalities in CBS, aiding in differential diagnosis and monitoring disease progression [115].

Recent studies have highlighted the complementary value of combining tau PET with FDG-PET in these disorders. For instance, a study by Liang et al. emphasized the utility of this combined approach in distinguishing corticobasal degeneration from other neurodegenerative conditions, underscoring the importance of integrating structural and functional imaging to enhance diagnostic precision [114].

Furthermore, recent advances in MRI have explored the use of neural probes to investigate microstructural and biochemical changes in PSP and CBS. Techniques including diffusion tensor imaging and magnetic resonance spectroscopy enable the assessment of white matter integrity, iron deposition, and neurochemical alterations [116,117]. These MRI-based markers provide valuable insights into disease mechanisms, while also holding promise as diagnostic and progression-monitoring tools in PSP and CBS. Moreover, quantitative susceptibility mapping (QSM), an advanced MRI technique, has become instrumental in identifying abnormal iron deposition within the brain, offering insights into the pathophysiology of neurodegenerative disorders [10]. In PSP, QSM studies consistently reveal increased magnetic susceptibility in subcortical regions, notably the globus pallidus, substantia nigra, and red nucleus [118]. Additionally, Kawabata et al. observed distinctive deformation of the red nucleus in PSP patients, detectable via coronal QSM imaging, which may serve as a morphological marker for the disease. Moreover, combining QSM measurements with volumetric data from the subthalamic nucleus has shown promise in differentiating PSP from PD, even in the early stages [119]. CBS also exhibits unique QSM characteristics. Miyata et al. reported asymmetric increases in susceptibility within the cerebral gyri of CBS patients, a pattern not typically observed in other neurodegenerative disorders. This “three-layer” appearance, particularly evident in the left precentral gyrus, suggests a cortical involvement that may aid in distinguishing CBS from other conditions [120]. Furthermore, QSM findings in CBS patients have demonstrated the potential to complement other imaging modalities, including FDG-PET, enhancing diagnostic accuracy and providing a more comprehensive understanding of the disease’s progression [121].

Lastly, we would like to note that, as this is a narrative review, the reproducibility of the results presented is limited. A future systematic review on this topic would greatly enhance the scientific validity of the evidence.

## 4. Materials and Methods

### 4.1. Search Strategy

We searched the PubMed database from its inception until 5 July 2025, using the algorithm “fluorodeoxyglucose positron emission tomography AND (progressive supranuclear palsy OR corticobasal)”, according to the Preferred Reporting Items for Systematic Reviews and Meta-Analyses guidelines [122]. Studies were included if they met the following criteria: (a) original research articles; (b) full text available in English; (c) human studies; (d) reporting the number of patients with PSP and CBS that were included in the study; and (e) investigations assessing the utility of FDG-PET for diagnosis or differential diagnosis from other neurodegenerative conditions. Narrative reviews, systematic reviews, meta-analyses, case reports, brief communications, opinion papers, letters to the editors, etc., were excluded from the study.

### 4.2. Study Selection and Categorization

**Figure 3:**
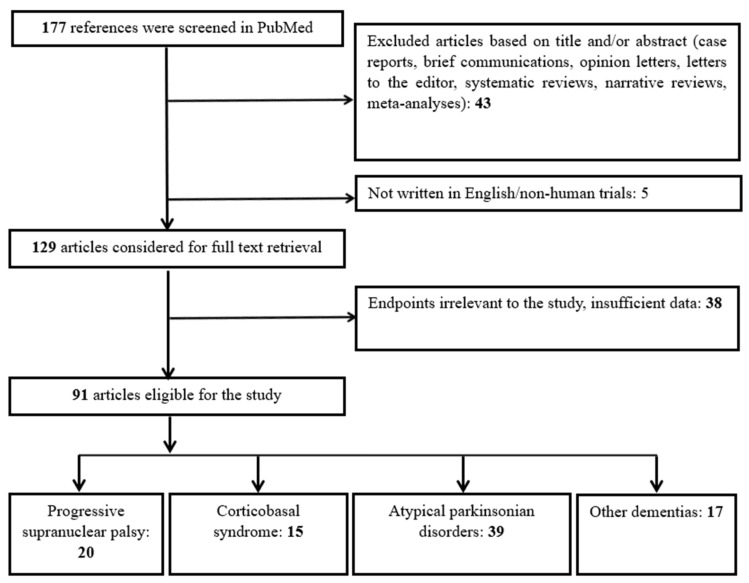
Flowchart of study selection and categorization.

As depicted in Figure 3, our algorithm initially yielded 177 results. Five studies were excluded due to being either non-English or not involving human subjects. Forty-three articles were excluded as they were reviews, meta-analyses, case reports, opinion papers, or editorials. This left 129 studies for the full-text review. Following this, 38 studies were excluded for being irrelevant to the purpose of our review. Ultimately, 91 studies were eligible. These 91 studies were categorized as follows: (a) 20 studies included only patients with PSP, (b) 15 studies included only patients with CBS, (c) 39 studies involved patients with APDs, including subgroups of PSP and/or CBS, and (d) 17 studies compared patients with PSP and/or CBS to individuals with FTD, AD, or other dementias.

## 5. Conclusions

In conclusion, FDG-PET may hold potential for improving the differential diagnosis of PSP and CBS, as they achieve high specificity with the aid of computational analysis but generally exhibit low sensitivity. However, future studies should focus explicitly on PSP and CBS rather than using them as comparative references for other neurodegenerative disorders. Large-scale, multicenter cohort studies employing standardized methodologies are necessary to enhance validity and reproducibility. Given the frequent overlap in pathology between PSP and CBS, pathology-confirmed study cohorts should be prioritized. Implementing these strategies could improve diagnostic accuracy and deepen our understanding of these complex diseases, ultimately paving the way for effective treatments.

## Figures and Tables

**Table 1:** Studies analyzing FDG-PET ^1^ patterns in PSP ^2^.

Study	4Rtauopathy ^3^ and Number of Patients (n)	Comparison Groups	Autopsy—Number of Confirmed Cases (n)	Main Findings
Foster et al., 1988 [16]	PSP (14)	NC ^4^	1 (confirmed multiple system atrophy)	− Reduced glucose metabolism in the caudate, thalamus, pons, and cerebral cortex, but not in the cerebellum, compared to controls − Cortical hypometabolism more pronounced in the cortex compared to subcortical regions
Goffinet et al., 1989 [17]	PSP (9)	-	0	− Hypometabolism in the frontal cortex, especially motor and premotor areas
Karbe et al., 1992 [18]	PSP (9)	NC	0	− Hypometabolism in upper midbrain, lentiform, and caudate nucleus − Less marked hypometabolism in the frontal cortex
Zhao et al., 2020 [19]	PSP (19)	-	0	− Hypometabolism in the midbrain, caudate, frontal lobes, and cingulate gyri − 92.3% hypometabolism in the midbrain compared to 57.9% “hummingbird sign” in the MRI ^5^
Yamauchi et al., 1997 [20]	PSP (10)	NC	0	− Hypometabolism of the frontal cortex was associated with atrophy of the corpus callosum
Mishina et al., 2004 [21]	PSP (16)	NC	2	− Midbrain hypometabolism, independent of clinical deterioration
Takahashi et al., 2011 [22]	PSP (16)	NC	0	− Greater alterations in FDG-PET compared to MRI in the frontal lobes, thalami, and midbrain − Positive correlation between MMSE ^6^, FDG uptake, and gray and white matter volume of the frontal lobes
Zwergal et al., 2011 [23]	PSP (16)	NC	0	− Decreased glucose metabolism in the thalami correlated with fall frequency
Zwergal et al., 2013 [24]	PSP (12)	NC	0	− Gait impairment was correlated with hypometabolism in the prefrontal cortex, subthalamic nucleus, and pedunculopontine/cuneiform nucleus complex
Amtage et al., 2014 [25]	PSP (26)	-	0	− Bilateral anterior cingulate and right lingual gyrus hypometabolism was correlated with downward gaze palsy
Isella et al., 2022 [26]	PSP (31)	-	0	− Hypometabolism of the dominant superior frontal cortex associated with reduced phonemic fluency
Doll-Lee et, 2025 [27]	PSP (22)	NC	0	− Reduced FDG uptake in the left inferior frontal gyrus and right angular gyrus − Reduced FDG uptake correlated with decreased overall cognition in the right frontal eye field, list learning in the left frontal eye field, and verbal fluency in premotor and supplementary motor cortices
Buchert et al., 2023 [28]	PSP-RS ^7^ (21), non-PSP-RS (20)	NC	0	− Higher sensitivity and specificity with automatic compared to visual analysis
Garraux et al., 2001 [29]	PSP-RS (21), non-PSP-RS (20)	NC	0	− Similar AUC ^8^ for volumetric MRI and FDG-PET in both groups
Black et al., 2024 [30]	PSP-RS (59), PSP-P (22) ^9^, PSP-CBS (6) ^10^, PSP-SL (28) ^11^, PSP-F (8) ^12^, PSP-PGF ^13^ (8), PSP-OM ^14^ (2), PSP-PLS (5) ^15^, PSP-PI (5) ^16^	-	43 (28 diagnosed with PSP, 15 with other neurodegenerative diseases)	− Frontal hypometabolism in 100% PSP-SL, PSP-CBS, and PSP-F, with lower rates in other subtypes − Hypometabolism asymmetric in PSP-CBS, PSP-SL, PSP-P, and PSP-F − FAB ^17^ scores negatively correlated with glucose metabolism
Hosaka et al., 2002 [31]	PSP (21), CBD ^18^ (22)	NC	0	− More prominent hypometabolism in the midbrain, anterior cingulate, and orbitofrontal cortex for PSP, and supplementary motor cortex and inferior parietal cortex for CBD
Juh et al., 2005 [32]	PSP (8), CBD (8)	NC	0	− Hypometabolism in the midbrain, thalamus, orbitofrontal, middle frontal cortex, and cingulate for PSP− Hypometabolism asymmetrically in the frontal, parietal, and cingulate for CBD
Amtage et al., 2014 [33]	PSP (12), CBS ^19^ (12)	NC	0	− Hypometabolism in midbrain, basal ganglia, and frontal cortex for PSP − Hypometabolism in parietal cortex for CBS
Zalewski et al., 2014 [34]	PSP (7), CBD (1)	-	8	− Hypometabolism in the frontal cortex for PSP − Hypometabolism in parietal cortex for CBS − Hypometabolism in the contralateral thalamus, middle cingulate gyrus, and sensorimotor cortex for PSP with unilateral motor impairment

^1^ FDG-PET: fludeoxyglucose-18–positron emission tomography, ^2^ PSP: progressive supranuclear palsy, ^3^ 4R: four-repeat, ^4^ NC: normal controls, ^5^ MRI: magnetic resonance imaging, ^6^ Mini-Mental State Examination, ^7^ PSP-RS: PSP–Richardson syndrome, ^8^ AUC: area under the curve, ^9^ PSP-P: PSP with predominant Parkinsonism, ^10^ PSP-CBS: PSP with predominant corticobasal syndrome, ^11^ PSP-SL: PSP with predominant speech/language impairment, ^12^ PSP-F: PSP with predominant frontal presentation, ^13^ PSP-PGF: PSP with progressive gait freezing, ^14^ PSP-OM: PSP with predominant ocular motor dysfunction, ^15^ PSP-PLS: PSP–primary lateral sclerosis, ^16^ PSP-PI: PSP with postural instability, ^17^ FAB: Frontal Assessment Battery, ^18^ CBD: corticobasal degeneration, ^19^ CBS: corticobasal syndrome.

**Table 2:** FDG-PET ^1^ of the main hypometabolic regions in PSP ^2^.

Study	Caudate Nucleus	Putamen	Globus Pallidus	Thalamus	Pons	Midbrain	Superior Cerebellar Peduncle	Cerebellum	Frontal Cortex	Parietal Cortex	Temporal Cortex	Occipital Cortex	Cingulate	Insular Cortex	Hippocampus
Foster et al., 1988 [16]	+	+	-	+	+	-	-	-	-	-	-	-	-	-	-
Goffinet et al., 1989 [17]	+	+	+	+	-	-	-	+	+ (motor/premotor)	-	-	-	-	-	-
Karbe et al., 1992 [18]	+	+	-	-	-	+	-	-	+ (sensorimotor)	+ (sensorimotor)	-	-	-	-	-
Zhao et al., 2020 [19]	+	+	-	-	-	+	-	-	+ (medial)	-	-	-	-	-	-
Yamauchi et al., 1997 [20]	-	-	-	-	-	-	-	-	+ (dorsolateral/medial)	-	-	-	-	-	-
Mishina et al., 2004 [21]	+ (head)	+ (anterior)	+ (anterior)	-	-	+	-	-	+ (superior/inferior/medial)	-	-	-	-	+	-
Takahashi et al., 2011 [22]	-	-	-	-	-	+ (tegmentum)	-	-	+	-	-	-	-	-	-
Zwergal et al., 2011 [23]	-	-	-	+	-	+ (tegmentum)	-	-	+ (middle)	-	-	-	+ (anterior)	-	-
Zwergal et al., 2013 [24]	-	-	-	+	-	+	-	+ (vermis)	+ (middle/inferior)	-	-	-	+ (anterior)	-	-
Amtage et al., 2014 [25]	-	-	-	-	-	-	-	+ (vermis)	+ (frontal eye filed)	+ (lingual/inferior)			+ (anterior)		
Isella et al., 2022 [26]	+	+	+	-	-	+	-	-	+ (dorsolateral/mesial prefrontal/inferior frontal)	-	-	-	-	-	-
Doll-Lee et, 2025 [27]	-	-	-	-	-	-	-	-	+ (inferior)	+ (angular)	-	-	-	-	-
Garraux et al., 2001 [29]	-	-		-	-	+	+	-	+ (orbitofrontal)	-	-	-	+ (anterior)		-
Black et al., 2024 [30]	-	-	-	-	-	-	-	-	+ (premotor/prefrontal/sensorimotor)	+ (sensorimotor)	-	-	-	-	-
Hosaka et al., 2002 [31]	+	-	-	-	-	+	-	-	+ (inferior)	-	-	-	+ (anterior)	-	-
Juh et al., 2005 [32]	-	-	-	+	-	+	-	-	+ (orbitofrontal/middle)	-	-	-	+	-	-
Amtage et al., 2014 [33]	-	-	-	+	-	-	-	-	+ (medial/sensorimotor)	+ (sensorimotor)	-	-	+ (middle)	-	-
Zalewski et al., 2014 [34]	+	-	-	+	-	+	-	-	+ (premotor/supplementary motor/precentral)	-	-	-	-	-	-

^1^ FDG-PET: fludeoxyglucose-18–positron emission tomography, ^2^ PSP: progressive supranuclear palsy.

**Table 3:** Studies analyzing FDG-PET ^1^ patterns in CBS ^2^.

Study	4Rtauopathy ^3^ and Patient Numbers (n)	Comparison Groups	Autopsy—Number of Confirmed Cases (n)	Main Findings
Blin et al., 1992 [36]	CBD (5) ^4^	NC ^5^	0	− Hypometabolism in the sensorimotor and temporal cortex contralateral to the most affected side
Klaffke et al., 2006 [37]	CBD (8)	-	0	− Asymmetrical hypometabolism in multiple cortical and subcortical areas − Only one patient with reduced binding in IBZM ^6^
Turaga et al., 2012 [38]	CBS (17)	-	0	− Asymmetrical hypometabolism of the left basal ganglia, inferior parietal, temporal, and frontal lobe correlated with neurocognitive deficits and MRI ^7^ atrophy
Franceschi et al., 2020 [39]	CBD (12)	-	0	− Asymmetrical hypometabolism of the contralateral hemisphere or, less commonly, bilateral hypometabolsim of the sensorimotor cortex
Sha et al., 2015 [40]	CBS (25)	-	8	− Higher sensitivity and lower specificity compared to amyloid PET ^8^ for diagnosing CBS
Mille et al., 2016 [41]	CBS (34)	-	0	− Hypometabolism in the contralateral frontal and parietal association areas and putamen − Only slightly asymmetrical presynaptic binding in DAT-SPECT ^9^
Isella et al., 2018 [42]	CBD (35)	-	0	− Hypometabolism in the left inferior parietal, primary motor, primary sensory, and insular cortices, associated with cognitive reserve
Isella et al., 2024 [43]	CBS (49)	-	0	− Hypometabolism of the left hemisphere associated with greater digit span deficits − Hypometabolism of the right hemisphere associated with greater visuospatial deficits
Pardini et al., 2019 [44]	CBS (29)	NC	29	− Distinct hypometabolic patterns may help differentiate the underlying pathology
Jo et al., 2021 [45]	CBD (52)	NC	0	− Hypometabolism of the angular gyrus in the ideomotor apraxia subtype − Hypometabolism of the precentral gyrus, precuneus, and posterior cingulate with the imitation apraxia subtype − Hypometabolism of the caudate and frontal cortex, regardless of the apraxia subtype
Parmera et al., 2021 [46]	CBS (31)	-	0	− Hypometabolism of the premotor and inferior frontal cortex correlated with dysarthria − Positive correlation of glucose uptake in the lateral temporal lobe and semantic fluency − Positive correlation of glucose uptake in the frontal operculum and inferior and middle temporal lobe
Parmera et al., 2024 [47]	CBS (32)	-	0	− Hypometabolism of the supplementary motor area, the bilateral striatum, and the anterior cingulate in the group that fulfilled the MDS ^10^ 4R criteria
Parmera et al., 2020 [48]	CBS (45)	-	0	− 76.92% sensitivity, 100% specificity, and positive predictive value for FDG-PET classification to detect positive amyloid PET scans
Nakano et al., 2022 [49]	CBS (16)	NC	1 (biopsy-confirmed)	− Hypometabolism of the precentral gyrus and thalamus for patients with negative amyloid PET and positive tau PET compared to NC
Ghirelli et al., 2025 [50]	CBS (33)	-	0	− More marked and asymmetrical hypometabolism of the temporal, parietal, and occipital area for patients with positive amyloid PET and positive tau PET

^1^ FDG-PET: fludeoxyglucose-18–positron emission tomography, ^2^ CBS: corticobasal syndrome, ^3^ 4R: four-repeat, ^4^ CBD: corticobasal degeneration, ^5^ NC: normal controls, ^6^ IBZM: ^123^I-iodobenzamide, ^7^ MRI: magnetic resonance imaging, ^8^ PET: positron emission tomography, ^10^ MDS: Movement Disorders Society.

**Table 4:** FDG-PET ^1^ of the main hypometabolic regions in CBS ^2^.

Study	Caudate Nucleus	Putamen	Thalamus	Midbrain	Cerebellum	Frontal Cortex	Parietal Cortex	Temporal Cortex	Occipital Cortex	Cingular Cortex	Insular Cortex	Hippocampus
Blin et al., 1992 [36]	+ C ^3^	+ C	+ C	-	-	+ C (sensorimotor)	+ C (associative)	+ C	-	-	-	-
Klaffke et al., 2006 [37]	-	-	+ C	-	-	+ C	+ C	-	-	-	-	-
Turaga et al., 2012 [38]	+ C	+ C	+ C	-	-	+ C (dorsolateral/prefrontal)	+ C (inferior)	+ L ^4^	-	-	-	-
Franceschi et al., 2020 [39]	+ C	+ C	+ C	-	-	+ (sensorimotor)	+ C (postcentral/precuneus)	-	+ C	-	-	-
Sha et al., 2015 [40]	+ B ^5^	-	-	-	-	+ B (sensorimotor)	+ B	+ B	-	-	-	-
Mille et al., 2016 [41]	+ C	+ L	+ B	-	-	+ C (precentral/middle)	+ C (postcentral/paracentral lobule)	-	-	+ C	-	-
Isella et al., 2018 [42]	-	-	-	-	-	+ L (precentral/pars triangularis)	+ L (postcentral/supramarginal)	+ L (postcentral/superior)	-	-	+ L (posterior)	-
Pardini et al., 2019 [44]	+ C	-	+ C	-	-	+ C (precentral/superior/middle/inferior)	+ C (postcentral/superior parietal/supramarginal)	-	-	-	-	-
Jo et al., 2021 [45]	+ C	-	-	-	-	+ C (superior/middle/precentral)	+ C (postcentral/angular/precuneus)	-	-	+ (anterior/posterior)	+	-
Parmera et al., 2021 [46]	+ B	+ B	+ B	-	-	+ L (premotor/inferior)	+ L (inferior) + B (prefrontal)	+ B (posterior)	+ (posterior)	+ B (posterior)	-	-
Parmera et al., 2024 [47]	+ C	+ C	+ C	-	-	+ C (supplementary motor/middle/inferior/prefrontal)	+ C (superior)	+ C (posterior)	-	+ C (anterior/middle/posterior)	-	-
Parmera et al., 2020 [48]	-	-	+ C	+ B	-	+ C (supplementary motor)	+ C (angular/superius parietal/paracentral lobule)	+ B (posterior superior/middle/inferior/fusiform)	-	-	-	-
Nakano et al., 2022 [49]	-	-	+ C	-	-	+ (precentral)	-	-	-	-	-	-
Ghirelli et al., 2025 [50]	-	-	-	-	-	+ C (middle)	+ B (postcentral/superior) + C (inferior/medial)	+ C (lateral)	+ C (lateral)	-	-	-

^1^ FDG-PET: fludeoxyglucose-18–positron emission tomography, ^2^ CBS: corticobasal syndrome, ^3^ C: contralateral to the most affected side, ^4^ L: left, ^5^ B: bilateral.

**Table 5:** Studies analyzing FDG-PET ^1^ patterns in PSP ^2^ and/or CBS ^3^ and Parkinsonian syndromes.

Study	4Rtauopathy ^4^ and Number of Patients (n)	Comparison Groups	Autopsy—Number of Confirmed Cases (n)	Main Findings
Eckert et al., 2008 [52]	PSP (21)	MSA ^5^, NC ^6^	0	− Decreased brainstem and bilateral medial frontal cortex metabolism − Decreased putamen and cerebellum metabolism in MSA − Assessments of pattern expression accurately discriminated patients from NC
Mudali et al., 2015 [53]	PSP (17)	PD ^7^, MSA, NC	0	− Moderate accuarcy for discriminating PSP from NC (AUC ^8^ 0.8) and relatively low accuracy from PD (AUC 67.6) and MSA (AUC 68.4)
Srulijes et al., 2012 [54]	PSP-RS (11) ^9^, PSP-P (8) ^10^	PD, NC	0	− Thalamic hypometabolism in PSP-RS − Putaminal hypometabolism in PSP-P − Putamen/thalamus uptake ratio effectively differentiated PSP-P from PSP-RS with good accuracy (AUC = 0.86) and from PD with an AUC of 0.80
Eckert et al., 2005 [55]	PSP (20), CBD (11) ^11^	PD, MSA	2 (MSA)	− Computer assessment agreed with clinical diagnosis in 92.4% of cases
Botha et al., 2014 [34]	PSP (28), CBS (26)	MSA	3 (PSP)	− High specificity (100%) and low sensitivity (29%) for pimple sign
Niethammer et al., 2014 [56]	PSP (51), CBD (27)	MSA, NC	3 (CBD)	− 24% overlap between CBD for asymmetric hypometabolic pattern − High specificity for CBD (92.7%) and PSP (94.1%) utilizing a logistic algorithm
Hellwig et al., 2014 [57]	PSP (7), CBD (5)	PD, MSA	0	− 90% diagnostic accuracy for differentiating between PSP, CBD, PD, and MSA
Tripathi et al., 2013 [58]	PSP (30), CBS (2)	PD, MSA	0	− High accuracy for clinical diagnosis among groups (90.4% for PD, 80% for MSA, 93.3% for PSP, and 100% for CBS)
Hellwig et al., 2015 [59]	PSP (22), CBD (8)	PD, DLB ^12^, MSA	0	− Moderate specificity and high sensitivity in diagnosing PSP (74%/95%) and CBD (75%/92%, respectively)
Tang et al., 2010 [60]	PSP (37)	PD, MSA	2 PSP, 1 MSA (CBD at autopsy)	− 88% sensitivity and 94%specificity for diagnosing PSP
Juh et al., 2004 [61]	PSP (7)	PD, MSA, NC	0	− Reduced metabolic activity in the neocortex for all disease groups − PSP hypometabolism in caudate nucleus, thalamus, midbrain, and anterior cingulum
Garraux et al., 2013 [62]	PSP (26), CBS (21)	PD, MSA	0	− Low classification accuracy for all groups
Tripathi et al., 2016 [63]	PSP (30)	PD, MSA	26	− 94% specificity for differentiating PSP from PD and MSA
Brajkovic et al., 2017 [64]	PSP, CBS (21)	PD, MSA	0	− 97% confirmed diagnosis in PSP and 100% in CBS
Marti-Andres et al., 2020 [65]	PSP-RS (47), PSP-P (18), PSP-PGF (8) ^13^	PD, NC	0	− PSP distinguished from NC with 80% sensitivity and 96.9% specificity and from PD with 80.4% sensitivity and 90.7% specificity − PSP-RS and PSP-P exhibited PSP-like hypometabolism more often than PD
Shen et al., 2020 [66]	PSP (34)	PD, MSA, NC	0	− Successful discrimination between all groups
Amod et al., 2022 [67]	PSP (11), CBS (2)	PD, DLB, MSA, NC	0	− 90% accuracy 90% of PD patients and 93% of patients with PSP and CBS
Tomse et al., 2022 [68]	PSP (11)	PD, MSA-P ^14^, NC	0	− PSP- and MSA-related patterns were highly sensitive and specific (AUC 0.99 and 0.96, respectively)
Eidelberg et al., 1991 [69]	CBD (5)	PD, NC	1	− Asymmetric hypometabolism of the inferior parietal lobule, thalamus, and hippocampus
Karbe et al., 1992 [70]	PSP (9)	PD, PD plus syndrome	0	− Hypometabolism of the brainstem, putamen, caudate, and frontal cortex
Klein et al., 2005 [71]	PSP (10)	PD	1	− Hypometabolism of the midbrain and anterior cingulate for the PSP group − Hypometabolism of the lateral visual cortex and right fusiform gyrus for the PSP group
Herting et al., 2007 [72]	PSP (9)	MSA, NC	0	− Hypometabolism of the bilateral frontal, right thalamus, and midbrain for the PSP group − Reduced glucose uptake of the dorsolateral prefrontal cortex correlated with depression severity for both PSP and MSA groups
Park et al., 2009 [73]	PSP (14)	PD, PAGF ^15^, NC	0	− Midbrain hypometabolsim for both PSP and PAGF − Frontal hypometabolism for PSP
Hellwig et al., 2012 [74]	PSP (20), CBD (9)	PD, PDD ^16^, DLB, MSA	0	− 74% sensitivity and 95% specificity for PSP − 75% sensitivity and 92% specificity for CBD
Teune et al., 2013 [75]	PSP (17)	PD, MSA, NC	0	− Highly discriminative metabolic patterns for all patient groups
Akdemir et al., 2014 [76]	PSP (4), CBD (2)	PD, DLB, MSA	0	− Hypometabolism in the basal ganglia for the majority of patients, usually asymmetric for PSP and PD − Hypometabolism of the thalami only in certain PSP and CBD cases
Baudrexel et al., 2014 [77]	PSP (8)	PD, MSA-P, NC	0	− Hypometabolism and increased mean diffusivity of the posterior putamen yielded similar efficacy in discriminating MSA-P from PSP, PD, and NC
Josephs et al., 2014 [78]	PSP (5)	PPAOS ^17^	0	− Hypometabolism pattern progressed to all subject during the study
Ko et al., 2017 [79]	PSP (38), CBS (42)	PD, DLB, MSA	0	− Metabolic and DAT-SPECT ^18^ binding patterns correlated in CBS and PD, not in PSP and MSA
Ge et al., 2018 [80]	PSP (20)	PD, MSA, NC	0	− Hypometabolism of the midbrain, ventrolateral and middle prefrontal cortex, cingulate, striatum and thalami, along with hypermetabolism in the hippocampus, insular, and temporoparietal regions effectively discriminated PSP from other study groups with high reproducibility.
Arnone et al., 2022 [81]	PSP (10), CBS (8)	PD, MSA	0	− Hypometabolic and hypermetabolic pattern maps improved diagnostic accuracy for non-experts for PSP, CBS, PD, and MSA − Only hypometabolic pattern maps improved diagnostic accuracy for experts for PSP, CBS, PD, and MSA
Lu et al., 2023 [82]	PSP (155)	PD, MSA	0	− 96.3%, 93.1%, and 94.8% diagnostic accuracy for PSP, PD, and MSA with age- and-gender-specific Z-score for brain metabolism, respectively
Ouartassi et al., 2023 [83]	PSP (12), CBS (27)	PD, NC	0	− Low rates of CBD-like hypometabolic pattern in the CBS group
Ali et al., 2024 [84]	PSP-RS (60), other PSP subtypes (51)	-	0	− Both FDG-PET and volumetric MRI are better at predicting the presence of a clinical feature than its severity − FDG-PET better for cortical-related and volumetric MRI better for subcortical-related features
Du et al., 2024 [85]	PSP (15)	PD, MSA-P, VP ^18^	0	− 0.968 AUC for distinguishing PD from PSP
Ling et al., 2024 [86]	PSP (234)	PD, MSA, NC	0	− 91.2% sensitivity for PSP with a radiomics-guided deep learning model
Ling et al., 2025 [87]	PSP (234)	PD, MSA, NC	0	− Disease-specific hypometabolism of the midbrain and midbrain-prefrontal disconnection for the PSP group
Pillai et al., 2025 [88]	PSP-RS (32), PSP-P (35), PSP-PI ^19^ (19), PSP-CBS ^20^ (4), PSP-SL ^21^ (3), PSP-OM ^22^ (1)	PD	0	− FDG-PET distinguished PSP from PD
Stokelj et al., 2025 [89]	PSP (37)	PD, MSA	0	− 0.95 AUC for PSP − 77%, 6%, and 18% of PSP patients correctly classified, misclassified, or undetermined with the proposed algorithm, respectively

^1^ FDG-PET: fludeoxyglucose-18–positron emission tomography, ^2^ PSP: progressive supranuclear palsy, ^3^ CBS: corticobasal syndrome, ^4^ 4R: four-repeat, ^5^ MSA: multiple system atrophy, ^6^ NC: normal controls, ^7^ PD: Parkinson’s disease, ^8^ AUC: area under the curve, ^9^ PSP-RS: PSP–Richardson syndrome, ^10^ PSP-P: PSP with predominant Parkinsonism, ^11^ CBD: corticobasal degeneration, ^12^ DLB: dementia with Lewy bodies, ^13^ PSP-PGF: PSP with progressive gait freezing, ^14^ MSA-P: MSA—Parkinsonian type, ^15^ PAGF: pure akinesia with gait freezing, ^16^ PDD: Parkinson’s disease dementia, ^17^ PPAOS: primary progressive apraxia of speech, ^18^ VP: vascular Parkinsonism, ^19^ PSP-PI: PSP with postural instability, ^20^ PSP-CBS: PSP with predominant corticobasal syndrome, ^21^ PSP-SL: PSP with predominant speech/language impairment, ^22^ PSP-OM: PSP with predominant ocular motor dysfunction.

**Table 6:** Studies analyzing FDG-PET ^1^ patterns in PSP ^2^ and/or CBS ^3^ and frontotemporal syndromes.

Study	4Rtauopathy ^4^ and Number of Patients (n)	Comparison Groups	Autopsy—Number of Confirmed Cases(n)	Main Findings
Cerami et al., 2016 [91]	PSP, CBD ^5^	nfvPPA ^6^, svPPA ^7^, lvPPA ^8^	0	− Asymmetric parietal hypometabolism in cases later diagnosed with CBD. − Patients meeting PSP criteria involved the midbrain and cerebellum.
Cerami et al., 2020 [92]	CBS	AD ^9^	0	− Positive CSF ^10^ biomarkers for AD correlated with AD hypometabolism. − Negative biomarkers correlated with bilateral frontoinsular and basal ganglia hypometabolism.
Caminiti et al., 2018 [14]	PSP, CBD	MCI ^11^, AD, FTD ^12^, DLB ^13^, bvFTD ^14^, PCA ^15^	0	− 50 out of 80 MCI cases progressed to dementia, including 2 to CBD and 1 to PSP.
Bergeron et al., 2020 [93]	CBS	AD, bvFTD, nfvPPA, svPPA, lvPPA, PCA	0	− Posterior cingulum was not the region with the most pronounced hypometabolism in any non-amnestic AD variants. − The angular gyrus showed the most hypometabolism in CBS associated with AD pathology.
Heikkinen et al., 2022 [94]	PSP, CBD	bvFTD	0	− bvFTD patients had hypometabolism in the left temporal lobe, superior cerebellar peduncle, cerebellar lingula and frontal lobes. − No cerebellar hypometabolism in PSP or CBD.
Isella et al., 2022 [95]	PSP, CBS	AD, PPA ^16^, PCA, DLB, bvFTD	0	− Cases with asymmetric temporoparietal profile and CBS or PPA phenotype were more frequently classified as AD-like, even without other AD biomarkers.
Teune et al., 2010 [96]	PSP (17), CBD (10)	PD ^17^, DLB ^18^, MSA ^19^, AD, FTD	0	− Hypometabolism of the prefrontal cortex, caudate, thalamus, and midbrain for PSP. − Hypometabolism of the contralateral cortex for CBD.
Wenzel et al., 2010 [97]	CBD (4)	AD, DLB, FTD, NC ^20^	0	− Insignificant effect of the stereotactic normalization on expert-based classification outcomes.
Garibotto et al., 2013 [98]	CBD (6)	AD, DLB, FTD, PDD ^21^	0	− 85.2% and 55.6% accuracy for FDG-PET, classified by discriminant analysis and cross-validation, respectively. − 100% and 88.9% of accurate classifications for FDG-PET and DAT-SPECT ^22^ combined.
Taswell et al., 2015 [99]	CBS (14)	AD, nfvPPA, svPPA, lvPPA	0	− 84% diagnostic accuracy for AD with FDG-PET. − 65–67% diagnostic accuracy for AD with clinical assessment.
Franceschi et al., 2020 [100]	CBD (12)	AD, DLB, FTD	0	− 0.58 (*p* < 0.05) Pearson correlation coefficient between metabolic z-score and lobar volume in the superior parietal lobule for CBD.
Franceschi et al., 2020 [101]	CBD (3)	AD, lvPPA, svPPA, bvFTD	0	− 3 out of 10 patients with crossed cerebellar diaschisis had imaging findings suggestive of CBD.
Franceschi et al., 2021 [102]	PSP (5), CBD (12)	bvFTD, lvPPA, svPPA, nfvPPA	0	− Hypometabolism in the posterior frontal cortex, thalamus, basal ganglia, and midbrain for PSP and asymmetric hypometabolism in the sensorimotor cortex, basal ganglia, and thalamus for CBD.
Joesphs et al, 2021 [103]	PSP (10), CBD (17)	TDP ^23^, AGD ^24^, Pick’s disease	32	− Insignificant metabolic differences at initial evaluation between patients with PSP or CBD − After four years, patients with CBD exhibited greater rates of metabolic decline and lower cortical metabolism compared to PSP.
Seckin et al., 2020 [104]	PSP (6), CBS (3)	-	0	− Slightly asymmetric, left hypometabolism for CBS. − Minimal or absent midbrain hypometabolism for PSP.
Gan et al., 2023 [105]	PSP (37), CBD (16)	AD, DLB, bvFTD, nfvPPA, FTD-ALS ^25^, VaD ^26^, MCI ^27^	0	− 62.5% of CBD and 56.8% of PSP patients exhibited a frontal and anterior temporal hypometabolic pattern.
Braun et al., 2025 [106]	PSP-RS ^28^ (18), PSP-P (7) ^29^, PSP-CBS ^30^ (3), PSP-F ^31^ (1), PSP-PGF ^32^ (2), PSP-PLS ^33^ (1), PSP-PI ^34^ (2), CBD (26)	AD, PD, DLB, MSA, TDP, PPAOS, PCA, AGD, Pick’s disease	137	− 0.91 diagnostic accuracy for PSP. − Most important objects for differentiating between the groups were in the midbrain, thalamus, and cerebellar dentate.

^1^ FDG-PET: fludeoxyglucose-18–positron emission tomography, ^2^ PSP: progressive supranuclear palsy, ^3^ CBS: corticobasal syndrome, ^4^ 4R: four-repeat, ^5^ CBD: corticobasal degeneration, ^6^ nfvPPA: nonfluent variant primary progressive aphasia, ^7^ svPPA: semantic variant primary progressive aphasia, ^8^ lvPPA: logopenic variant primary progressive aphasia, ^9^ AD: Alzheimer’s disease, ^10^ CSF: cerebrospinal fluid, ^11^ MCI: mild cognitive impairment, ^12^ FTD: frontotemporal dementia, ^13^ DLB: dementia with Lewy bodies, ^14^ bvFTD: behavioral variant frontotemporal dementia, ^15^ PCA: posterior cortical atrophy, ^16^ PPA: primary progressive aphasia, ^17^ PD: Parkinson’s disease, ^18^ DLB: dementia with Lewy bodies, ^19^ MSA: multiple system atrophy, ^20^ NC: normal controls, ^21^ PDD: Parkinson’s disease dementia, ^22^ DAT-SPECT: dopamine transporter single-photon emission computed tomography, ^23^ TAR DNA binding of 43 Kda, ^24^ AGD: argyrophilic grains disease, ^25^ FTD-ALS: frontotemporal dementia with amyotrophic lateral sclerosis, ^26^ VaD: vascular dementia, ^27^ MCI: mild cognitive impairment, ^28^ PSP-RS: PSP-Richardson syndrome, ^29^ PSP-P: PSP with predominant Parkinsonism, ^30^ PSP-CBS: PSP with predominant corticobasal syndrome, ^31^ PSP-F: PSP with predominant frontal presentation, ^32^ PSP-PGF: PSP with progressive gait freezing, ^33^ PSP-PLS: PSP–primary lateral sclerosis, ^34^ PSP-PI: PSP with postural instability.

## Data Availability

No new data was created during the preparation of this manuscript.

## Abbreviations

The following abbreviations are used in this manuscript:

4R	four-repeat
AD	Alzheimer’s disease
APD	atypical Parkinsonian disorder
AUC	area under the curve
bvFTD	behavioral variant frontotemporal dementia
CBD	corticobasal degeneration
CBS	corticobasal syndrome
CSF	cerebrospinal fluid
DaT-SPECT	dopamine transporter imaging with single-photon emission computed tomography
DLB	dementia with Lewy bodies
FAB	Frontal Assessment Battery
FDG-PET	fludeoxyglucose-18–positron emission tomography
FTD	frontotemporal dementia
GGT	globular glial tauopathy
IBZM	^123^I-iodobenzamide
lvPPA	logopenic variant PPA
MCI	mild cognitive impairment
MDS	Movement Disorders Society
MDS-UPDRS	MDS-Unified Parkinson’s Disease Rating Scale
MMSE	Mini-Mental State Examination
MoCA	Montreal Cognitive Assessment
MRI	magnetic resonance imaging
MSA	multiple system atrophy
MSA-P	multiple system atrophy-parkinsonian type
nfvPPA	nonfluent variant PPA
PAGF	pure akinesia with gait freezing
PCA	posterior cortical atrophy
PD	Parkinson’s disease
PDD	Parkinson’s disease dementia
PET	Positron emission tomography
PPA	primary progressive aphasia
PPAOS	primary progressive apraxia of speech
PSP	progressive supranuclear palsy
PSP-CBS	PSP with predominant corticobasal syndrome
PSP-F	PSP with predominant frontal presentation
PSP-OM	PSP with predominant ocular motor dysfunction
PSP-P	PSP with predominant Parkinsonism
PSP-PGF	PSP with progressive gait freezing
PSP-PI	PSP with postural instability
PSP-PLS	PSP–primary lateral sclerosis
PSP-SL	PSP with predominant speech/language impairment
PSP-RS	PSP–Richardson syndrome
ROI	region of interest
SIS	Saccadic Impairment Scale
SPECT	single-photon emission computed tomography
svPPA	semantic variant PPA
